# Structural and functional analysis of cystatin E reveals enzymologically relevant dimer and amyloid fibril states

**DOI:** 10.1074/jbc.RA118.002154

**Published:** 2018-07-02

**Authors:** Elfriede Dall, Julia C. Hollerweger, Sven O. Dahms, Haissi Cui, Katharina Häussermann, Hans Brandstetter

**Affiliations:** From the ‡ Department of Biosciences, University of Salzburg, A-5020 Salzburg, Austria and; the § Center for Integrated Protein Science Munich, Technical University of Munich, D-85748 Munich, Germany

**Keywords:** enzyme inhibitor, conformational change, amyloid, cysteine protease, protein stability, protein structure

## Abstract

Protein activity is often regulated by altering the oligomerization state. One mechanism of multimerization involves domain swapping, wherein proteins exchange parts of their structures and thereby form long-lived dimers or multimers. Domain swapping has been specifically observed in amyloidogenic proteins, for example the cystatin superfamily of cysteine protease inhibitors. Cystatins are twin-headed inhibitors, simultaneously targeting the lysosomal cathepsins and legumain, with important roles in cancer progression and Alzheimer's disease. Although cystatin E is the most potent legumain inhibitor identified so far, nothing is known about its propensity to oligomerize. In this study, we show that conformational destabilization of cystatin E leads to the formation of a domain-swapped dimer with increased conformational stability. This dimer was active as a legumain inhibitor by forming a trimeric complex. By contrast, the binding sites toward papain-like proteases were buried within the cystatin E dimer. We also showed that the dimers could further convert to amyloid fibrils. Unexpectedly, cystatin E amyloid fibrils contained functional protein, which inhibited both legumain and papain-like enzymes. Fibril formation was further regulated by glycosylation. We speculate that cystatin amyloid fibrils might serve as a binding platform to stabilize the pH-sensitive legumain and cathepsins in the extracellular environment, contributing to their physiological and pathological functions.

## Introduction

Cysteine proteases are key regulators in many physiological processes. Consequently, dysregulation of protease activity can have severe effects resulting in a variety of pathologies, including cancer and Alzheimer's disease (1, 2). Therefore, proteases must be regulated delicately and on different levels. Cystatins are inhibitors specifically controlling the activity of cysteine proteases. The cystatin superfamily shares a common fold that is characterized by a 5-stranded antiparallel β-sheet that is enwrapping a central α-helix. It is further organized into three subfamilies: (i) stefins; (ii) cystatins; and (iii) kininogens, fetuins, and noninhibitory cystatins (3, 4). Family 1 cystatins (stefins) are mainly localized intracellularly and ubiquitously expressed in most cell types (3, 5). They are potent inhibitors of papain (family C1) and the papain-like cathepsins with *K_I_* values in the low nanomolar range (6, 7). The interaction of stefins with papain is mediated by a tripartite wedge-shaped structure formed by the N terminus (Ser^1^–Val^10^, cystatin C numbering) and two hairpin loops (loops L1 and L2). Essentially, the N terminus binds to the nonprimed side, whereas the two adjacent hairpin loops occupy the primed substrate–binding sites.

Family 2 cystatins resemble the largest subfamily of the cystatin fold, with seven members identified so far. In contrast to the stefins, selected family 2 cystatins (C, E/M, and F) harbor, in addition to their papain-binding site, a legumain binding site (8–10). Human legumain is a caspase-like cysteine protease (family C13) that mainly localizes to the endo-lysosomal system, where it plays an important function for the processing of antigens for presentation on the MHCII complex (11). On a pathophysiological level, legumain has been implicated in various disorders, including cancers and Alzheimer's disease (12–14). Under these conditions, legumain was found translocated to the nucleus, to the cytoplasm, and extracellularly. Because of its strict specificity for cleaving after asparagine residues, it is synonymously referred to as the asparaginyl-endopeptidase (AEP)^2^ (15, 16). This strict preference is exploited by the legumain-inhibitory cystatins C, E, and F, which use a conserved Asn^39^ residue, localized on a reactive center loop different from the papain-inhibitory site to specifically bind to the legumain active site (9, 17). Furthermore, the interaction with legumain involves an additional legumain exosite loop (LEL) inserted between cystatin strands β3 and β4. Complex formation leads to conformational stabilization of the pH-sensitive legumain at near neutral pH. Unlike family 1 cystatins, legumain-inhibitory cystatins are secreted outside the cell and are in some cases glycosylated (10, 18–20). Whereas cystatin C is ubiquitously expressed in different human tissues, cystatin E/M is mainly localized to skin epithelia, emphasizing its role in cutaneous biology (5, 10, 21). Co-localization of human cystatin E (hCE) and legumain has been reported in hair follicles (22).

Cystatins not only encode a high intrinsic variability because of their function as dual protease inhibitors but also because of their ability to transform to distinct oligomerization states upon conformational destabilization. Factors trigging this oligomerization include N-terminal truncation by proteolytic enzymes, acidic pH, heating, and point mutations. These cause dimer formation via a domain-swapping mechanism (23–25). Essentially, the N-terminal segment, comprising β1, α, and β2 up to the L1 loop, of one monomer exchanges with that of a second monomer (26). Consequently, the papain-inhibitory site becomes inaccessible, whereas the legumain-inhibitory site remains intact. Cystatin C oligomerization leads to the formation of amyloid deposits in the brain at advanced age (25). A naturally occurring L68Q variant was identified in the cerebral fluid of patients suffering from hereditary cystatin C angiopathy (Iceland disease), which accelerates this process significantly (6, 27). Similarly, N-terminally truncated cystatin C, lacking the first 10 amino acids of the native sequence, was isolated from cystatin C amyloid deposits (28). This truncation was associated with proteolytic processing by proteases released to the cerebrospinal fluid and similarly results in accelerated formation of amyloid depositions (29). Stefin B was also reported to form amyloid fibrils and is an Aβ-binding protein and therefore supposed to play a role in Alzheimer's disease (30–32).

Both legumain and cystatins became attractive drug targets due to their relevance in different types of cancer and dementia. Among the cystatins, the family 2 cystatins became especially interesting, because of their function as dual protease inhibitors and because they are secreted to the extracellular space, where legumain and cathepsins are similarly observed under pathophysiologic conditions. Cystatin E is the most potent physiologic legumain inhibitor, binding 100-fold more tightly as compared with cystatin C (7). Thereby, it is associated with a tumor suppressor function in prostate cancer, melanoma, and oral carcinoma cells (33–35). Furthermore, cystatin E has been observed co-localized with legumain in the extracellular environment under normal and under pathophysiologic conditions (22, 36). Notably, not only co-localization but also co-trafficking of legumain together with cystatin E from outside the cell to inside a cell has been reported (37).

However, cystatin E's physico-chemical properties, including its propensity to form dimers and higher oligomers, have hardly been studied so far. Therefore, we set out to challenge the conformation of cystatin E by different triggering factors, including N-terminal truncation, pH, and heating. Furthermore, we analyzed the effect of these destabilizing factors on its three-dimensional structure and inhibitory function.

## Results

### Cystatin E forms a dimer under destabilizing conditions

**Figure 1.**
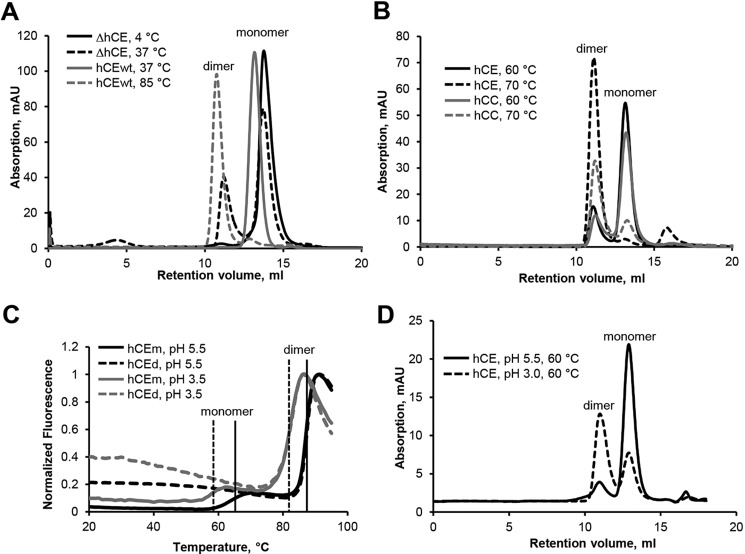
**Dimerization of hCE is triggered by destabilization.** *A*, dimer formation of N-terminally truncated (Δ*hCE*) and WT (*hCEwt*) hCE was assayed following incubation at the indicated temperatures using SEC. *B*, hCE and hCC were incubated at the indicated temperatures before injection onto the SEC column. At 70 °C, >90% conversion to the dimeric form could be observed. *C*, thermal denaturation curves were collected for monomeric (*hCEm*) and dimeric (*hCEd*) hCE at the indicated pH values. Melting Temperatures (*T_M_*) could be determined to be 65 °C (transition 1) and 87 °C (transition 2) at pH 5.5 for hCEm and hCEd, respectively. Acidic pH led to a reduction of *T_M_* (*dashed*, *vertical lines*). Transition 1 was only observed for monomeric hCE and corresponds to the conversion of monomeric to dimeric hCE. *D*, incubation of hCE at pH 3.0 led to more efficient conversion to the dimeric form. *mAU*, milli-absorbance units.

To mimic the effect of N-terminal proteolytic processing, we recombinantly produced an N-terminally truncated human cystatin E (ΔhCE) variant, lacking the first 10 amino acids following the mature N terminus (Δ(Arg^4^–Leu^13^)hCE; Δ*hCE* in Fig. S1). Size-exclusion experiments (SEC) revealed that whereas full-length WT hCE migrated at the expected elution volume of the monomeric 15-kDa protein, the N-terminally truncated ΔhCE variant showed an additional peak eluting at the size of a dimer (Fig. 1*A*). This observation was already the first indication for a similar tendency of hCE to oligomerize as described for other family members. As this oligomerization tendency correlated with conformational destabilizations, we next investigated the effect of heating on full-length hCE. Interestingly, we found that cystatin E monomer efficiently converted to a dimeric form upon incubation at ≥70 °C (Fig. 1, *A* and *B*). Using SEC, we could determine the transition temperature where 50% conversion was reached to be 65 °C (Fig. 1*B*). This was further cross-confirmed by ThermoFluor experiments (64), which revealed a melting temperature of monomeric hCE of 65 °C (Fig. 1*C*, *black curve*). In this particular case, thermally induced increase in fluorescence at 65 °C corresponded to the transition to dimeric hCE rather than to protein unfolding. To highlight the relevance of these experiments, we used cystatin C as a standard to compare the thermal energy barriers for dimer formation between the two family members. Significantly, cystatin C showed qualitatively and quantitatively the same behavior as hCE with 50% conversion to dimeric human cystatin C (hCC) at 65 °C and 100% conversion at 70 °C (Fig. 1*B*). Similarly, we tested the effect of pH on hCE dimerization. Indeed, incubation of monomeric hCE at pH 3.0 could significantly reduce the transition temperature. SEC experiments revealed efficient conversion to dimeric hCE already at 60 °C (Fig. 1*D*). Additionally, using the ThermoFluor method, we observed a reduction of the transition temperature by 8 °C upon incubation at pH 3.5 (Fig. 1*C*, *gray curve*).

### The cystatin E dimer is the thermodynamically preferred conformation

Based on these experiments, we concluded that conversion of monomeric to dimeric hCE is associated with an energy barrier that needs to be overcome. The energy barrier can be reduced by destabilizing conditions like N-terminal truncation, low pH, and heating. Similarly, long-term incubation at ambient temperature will have the same effect. To test whether the hCE dimer is a stable folding state by itself, we examined melting curves of monomeric and dimeric hCE (Fig. 1*C*). Dimeric hCE was generated by incubation of the monomer at 80 °C. Interestingly, the dimer showed an increased melting temperature, indicating that once the energy burden of dimerization has been overcome, the dimer is the structurally more stable conformation. Additionally, we found the monomer–dimer transition to be irreversible (*i.e.* dimeric hCE could not be converted back to monomeric hCE). Monomeric hCE thus represents a metastable state that is kinetically preferred in the folding process over the more stable dimeric hCE. Together, these observations were consistent with a cystatin C–like mode of dimerization, which is mediated by domain swapping (26).

### The cystatin E dimer forms via domain swapping

**Figure 2.**
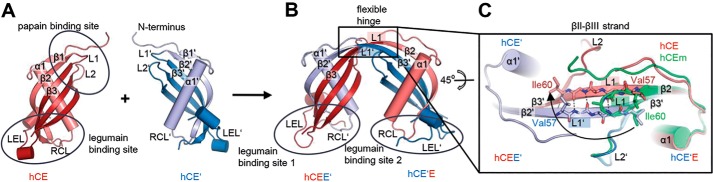
**The cystatin E dimer forms via domain swapping.** *A*, crystal structure of monomeric hCE (PDB entry 4N6L) with exposed papain- and legumain-binding sites. The regions of two monomers (*red* and *blue*) that undergo domain swapping are shown in *light colors. B*, crystal structure of the hCE dimer illustrated in a *cartoon representation*. The dimer is composed of two hCE monomers where the N-terminal region (*light blue*) of molecule hCE′ swapped out and integrated into the equivalent position on molecule hCE and vice versa. Thereby, two symmetric subunits, hCEE′ and hCE′E, are formed. *C*, *top view* of the flexible hinge region formed by the former L1 loops. Upon domain swapping, L1 (*light red*) and L1′ (*light blue*) rotated out by 90° and thereby formed the βII-βII strand connecting β2 to β3 and β2′ to β3′, respectively. The structure of monomeric hCE (*green cartoon*) was superposed onto the hCE′E subdomain.

To unveil the molecular mechanism of hCE dimerization, we determined the crystal structure of dimeric hCE, which was preincubated at 80 °C. Cystatin E crystals diffracted to 2.9 Å resolution and revealed dimer formation via domain swapping (Fig. 2 (*A* and *B*) and Table S1). The N-terminal region β1-α1-β2-L1 of one cystatin E molecule (hCE) swapped out and integrated at the equivalent position of a second cystatin E molecule (hCE′), and vice versa. To allow this subdomain movement, loop L1, which connects β-strands 2 and 3 in the monomer, adopted an extended conformation. Two elongated β-strands βII and βIII composed of the monomeric β2-L1-β3 of monomers A and B, respectively, aligned in an antiparallel fashion and thereby bridged the two monomers (Fig. 2 (*B* and C) and Fig. S2 (*A* and *B*)). The monomeric loops L1 and L1′ of either hCE are integrated within the resulting extended β-sheet, with the local symmetry dyad passing through them (Fig. 2*C*). Consequently, dimeric hCE has integrated four additional L1, L1′-derived hydrogen bonds, contributing to its higher thermal stability and energetically more stable conformation as compared with the monomeric structures (Fig. 2*C*). Additionally, the monomer–dimer transition is accompanied by a conformational relaxation of the strictly conserved Val^57^, relaxing the unfavorable monomer conformation, (ϕ ψ) = (−121°|−144°), to a favorable dimer conformation, (ϕ ψ) = (−140°|137°) (Fig. S1; PDB entry 4N6L) (38). Together, the L1–L1′ β-sheet formed a hinge region connecting two hCE monomers.

Analysis with PDBePISA (39) and PDBsum (40) revealed that the hCE dimer interface is built up by 45 hydrogen bonds, 361 nonbonded contacts, and two charge-driven interactions. This results in a buried surface area of the dimer interface of 6710 Å^2^ and an interface area of ∼3354 Å^2^. The high content in nonbonded contacts is also reflected in a very low-solvation free energy gain Δ*^i^G* upon complex formation of −43.5 kcal/m. Furthermore, the free energy of complex dissociation, Δ*G*^diss^, of 55.4 kcal/m indicates that the dimer is thermodynamically stable.

### The cystatin E dimer adopts a structurally distinct conformation

From a structural perspective, the hCE dimer was assembled from two hCE monomer conformers that are connected via their L1 loops. When we compared the structural similarity of the two half-domains hCEE′ and hCE′E within the dimer, we observed a relatively low root mean square deviation (RMSD) of the C^α^ positions (0.33 Å). The deviations between the two subdomains may relate to different packing environments within the crystal lattice. Interestingly, superposition of dimeric hCE with monomeric hCE revealed an average RMSD of 0.66 Å, indicating significant structural differences in hCE monomer as compared with the corresponding dimeric subdomain (Fig. 2*A*). Structural differences accumulated from a number of small rearrangements that accompanied the integration of the N-terminal β1-α1-β2 region from one molecule into the second. Therefore, the cystatin E monomer and dimer are two structurally distinct subspecies; the dimer is not the sole combination of two monomers.

When we superposed hCE and hCC monomer structures, we found an RMSD of the C^α^ positions of 1.3 Å (Fig. S2*C*). Interestingly, the difference between the hCE and hCC dimer structures is increased by 2-fold (RMSD = 2.8; Fig. S2*D*). Most of this high difference can be accounted for by the flexibility of the connecting hinge region (L1–L1′). Small structural changes within this region translate into large motions in the peripheral regions (*e.g.* the legumain-binding site). Given the flexibility of the connecting hinge, differences in crystal packing may also contribute to differences in the relative spatial orientation of the two subdomains, both in the hCE and hCC dimer structures (25). Therefore, we analyzed the crystal packing of the hCE dimer crystals. Remarkably, we discovered the assembly of two hCE dimers to a tetramer (Fig. S2*E*). A similar assembly was described previously for stefin B (PDB code 2OCT) and cystatin C (PDB code 1G96). The stefin B tetramer forms via a handshaking mechanism of the L2 loops that is triggered by a proline switch (Pro^105^ cystatin C numbering; Fig. S2*F*). Pro^105^ is conserved throughout the whole cystatin family. However, the L2 loop is shortened by 3 amino acids in the family 2 cystatins (Fig. S1). Hence, a direct handshake between two L2 loops is not possible. Nevertheless, the overall assembly (nearly parallel β2-β3 strands) is similar between stefin B, cystatin C, and cystatin E and therefore suggests a conserved mechanism of oligomerization. The dimers interact via hydrophobic contacts involving residues of the former L1 loops. The hCE tetramer is visible in the crystal structure but not in SEC experiments, indicating a low affinity of this assembly.

### The cystatin E dimer is a functional legumain inhibitor

**Figure 3.**
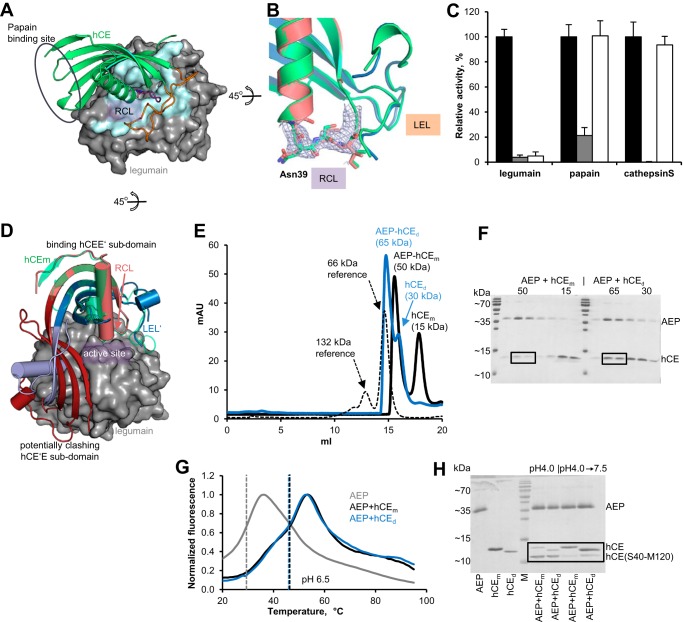
**The legumain-inhibitory site on dimeric hCE is accessible and fully functional.** *A*, crystal structure of the hCE–legumain complex (PDB code 4N6O). hCE is shown in a *green cartoon*, and the RCL harboring the P1-Asn^39^ residue is shown in *purple*, the LEL is shown in *orange*, and legumain is shown in *gray surface*. The contact area on legumain is shown in *light blue. B*, superposition of hCE monomer (*green*) and dimer (*red* and *blue*) in a *cartoon representation*. Intactness of the RCL was confirmed by the continuous electron density map (*F_o_* − *F_c_* omit map, contoured at 2.0 σ). *C*, inhibition of papain, cathepsin S, and legumain by monomeric (*gray*) and dimeric (*white*) cystatin E was assayed using fluorogenic FR-AMC (papain and cathepsin S) and Z-AAN-AMC (legumain) substrates. Activities were normalized to control reactions harboring enzyme only (*black*). *D*, model of a legumain–hCE dimer complex. Legumain is illustrated as a *gray surface*, dimeric hCE as a *blue-red cartoon*, and monomeric hCE as a *green cartoon*. Because hCEE′ and hCE′E are symmetric subdomains, they are in principle both capable of binding to the legumain active site. *E*, legumain (*AEP*) was incubated with monomeric (*hCE_m_*) and dimeric (*hCE_d_*) hCE, and complex formation was investigated via SEC. Buried surface area monomer (66 kDa) and dimer (132 kDa) peaks served as a reference (*black*, *dashed line*). *F*, SDS-PAGE of the peak fractions from the experiment described in *E*). *G*, thermal denaturation curves of legumain alone (*gray curve*) and precomplexed with monomeric hCE (*black curve*) and dimeric hCE (*blue curve*) were recorded at pH 6.5 following the ThermoFluor method. *Dashed lines*, melting temperatures (*T_M_*). *H*, SDS-PAGE showing legumain alone, monomeric hCE, dimeric hCE, molecular weight marker (*M*), and legumain incubated with monomeric hCE (*AEP* + *hCE_m_*) or dimeric hCE (*AEP* + *hCE_d_*) at pH 4.0 and after a subsequent shift to pH 7.5. Incubation of hCE with legumain at pH 4.0 leads to cleavage after P1-Asn^39^, which is visible in the (Ser^40^–Met^120^)-hCE band. Subsequent shift to neutral pH (7.5) led to conversion of cleaved hCE to intact hCE upon religation of the Ser^38^–Asn^39^ peptide bond on hCE. Dimeric hCE was prepared from N-terminally truncated hCE. *mAU*, milli-absorbance units.

The hCE dimer is a unique folding state that encodes two legumain-binding sites on one molecule. In contrast to monomeric hCE, the legumain-binding sites of the dimer are built up by contributions of both hCE chains (*e.g.* legumain-binding site 1 is built up from the RCL′ (reactive center loop) of one chain, hCE′, and the LEL of the second chain, hCE, and vice versa (Figs. 2*B* and 3*A*). Despite this large structural rearrangement, the local conformations of the legumain RCL and LEL were virtually identical in monomeric and dimeric hCE structures (Fig. 3*B*). Thus, we hypothesized that dimeric hCE is still functional as a legumain inhibitor. Indeed, when we incubated legumain with dimeric hCE, we observed a complete loss of its Ala-Ala-Asn-AMC substrate turnover (Fig. 3*C*). The crystal structure of dimeric hCE uncovered two symmetric and functional legumain-binding sites, either of which is accessible for legumain binding. To test whether a simultaneous binding of two legumain molecules by one hCE dimer (*i.e.* a tetrameric assembly) is sterically feasible, we prepared a model of such a complex based on the crystal structures of the hCE dimer and the legumain–hCE complex (PDB entry 4N6O (17)). First, we superposed the hCE dimer onto the legumain–hCE complex (Fig. 3*D*). We found virtually identical intermolecular contacts for either of the two possible docking modes (*i.e.* docking legumain to the hCEE′ or to the hCE′E submolecule was equally well accessible). However, from these docking models, it became immediately clear that simultaneous binding to both sites is sterically impossible. When legumain binds to the hCEE′ site, the free hCE′E site is in close proximity to the legumain insertion loop on legumain and vice versa. Hence, a trimer composed of one hCE dimer and one legumain (AEP) seemed to be the most likely assembly (Fig. 3*D*). To test this hypothesis, we performed size-exclusion experiments of a preformed legumain–hCE dimer complex. Indeed, we observed a peak at the size of a trimer, but not at the expected size of a tetramer (Fig. 3*E*). Additionally, SDS-PAGE revealed a 2-fold higher hCE concentration in the fractions of the legumain–hCE dimer complex (*AEP–hCE_d_*) as compared with legumain–hCE monomer complex (*AEP–hCE_m_*; Fig. 3*F*). The legumain concentration was identical in both experiments. The difference in hCE concentration within the fractions containing the hCE–legumain complex indicates a 2:1 stoichiometry rather than 2:2. Thus, cystatin E virtually loses 50% of its legumain binding sites via domain swapping.

The observation of symmetric legumain binding sites within the cystatin E dimer suggested that binding to both sites is possible at the same affinity. Furthermore, structurally identical binding sites in the monomer and dimer implied similar affinity constants toward legumain. Indeed, we could determine a *K_I_* of 10.7 ± 5.6 pm for monomeric hCE and 13.5 ± 6.7 pm for dimeric hCE. Molar concentrations of monomeric and dimeric hCE were calculated assuming molecular masses of 15 and 30 kDa, respectively. Although docking models of the hCE dimer–legumain complex showed some minor clashes between the unbound legumain binding site on the hCE dimer and legumain (Fig. 3*D*), these did not translate into weakened inhibition constants. (Mostly) unhindered complex formation is possible due to the flexible hinge region (L1–L1′).

Similar *K_I_* constants of dimeric *versus* monomeric cystatin E implied that the mode of binding of dimeric hCE to legumain is qualitatively similar to binding of monomeric hCE. To further test this conclusion, we investigated features that are characteristic for the hCE–legumain complex. First, we could previously show that interaction of the LEL on cystatin E with the legumain-primed substrate-binding site has a positive effect on its thermal stability at near neutral pH, where the isolated enzyme by itself is unstable (17, 41). The same is true for the legumain–hCE dimer complex (Fig. 3*G*). Second, we observed pH-dependent cleavage and religation of monomeric hCE at the conserved Asn^39^ residue located on the legumain reactive center loop of hCE by legumain (17). Cleavage happens at pH <5.5, and religation is observed upon incubation of cleaved hCE at pH >6.5. We observed the same pH-dependent behavior when dimeric hCE was used (Fig. 3*H*). Together, these observations are in nice agreement with the crystal structure of dimeric hCE that uncovered structurally and functionally uncompromised legumain-binding sites.

### The cystatin E dimer is not functional as papain inhibitor

In contrast to the legumain-inhibitory site, the papain-inhibitory site structurally differs in monomeric and dimeric hCE (Fig. 2, *A* and *B*). Upon dimerization, the L1 loop, which is essential for binding to the papain S1′ site, was incorporated into the newly formed βII-βIII sheet. Thus, loop L1 is not available for binding anymore, and the papain-inhibitory site is destroyed by the dimer interface. In agreement with this observation, dimeric hCE did not show inhibition of papain and human cathepsin S in a peptide substrate hydrolysis assay (Fig. 3*C*). Consequently, dimerization of cystatin E led to 100% loss of its cathepsin-inhibitory function.

### Glycosylation on the L2 loop is compatible with dimerization

**Figure 4.**
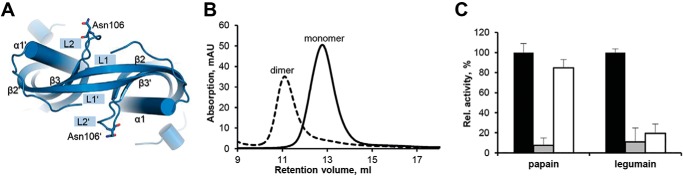
**Glycosylation is compatible with hCE dimerization.** *A*, *top view* of the hCE dimer structure in *cartoon representation*. The Asn^112^ residues located on the L2 loops that are prone to glycosylation are shown in *sticks. B*, glycosylated hCE produced in LEXSY was incubated at 20 °C (*black curve*) and 85 °C (*dashed*, *black curve*) for 10 min. Subsequently, both samples were injected onto an S75 10/300 GL column. Incubation at 85 °C led to a shift in the retention volume that corresponds to dimeric hCE. *C*, inhibition of papain and legumain by monomeric (*gray*) and dimeric (*white*) glycosylated cystatin E was assayed using fluorogenic FR-AMC (papain) and Z-AAN-AMC (legumain) substrates. Activities were normalized to control reactions harboring enzyme only (*black*). *mAU*, milli-absorbance units.

Native cystatin E harbors an *N*-glycosylation site on its L2 loop (Asn^112^). Because both glycosylated and nonglycosylated hCE were previously reported *in vivo*, we were interested in the relevance of glycosylation for hCE dimerization (20, 42). The crystal structure of the hCE dimer suggested no negative effect of glycosylation on dimer formation (Fig. 4*A*). To test whether this is the case, we incubated glycosylated hCE produced in the *Leishmania tarentolae* expression system (LEXSY) at 85 °C and subjected it to SEC. Indeed, we observed a shift corresponding to dimeric, glycosylated hCE (Fig. 4*B*). In agreement with the domain-swapping mechanism, this dimer similarly inhibited legumain but not papain in a fluorescent peptide substrate assays (Fig. 4*C*). Furthermore, ThermoFluor experiments of glycosylated hCE revealed a similar thermal unfolding behavior as observed for unglycosylated hCE (Fig. S3).

### Cystatin E is resistant to heterodimerization

Because the overall structures of stefin B and cystatin C and E dimers are superficially similar, we questioned whether dimerization might not be restricted to homodimer formation but rather open to the possibility of heterodimerization as well. To test this, we performed pulldown assays using a cystatin E construct carrying a C-terminal Strep-tag and WT cystatin C. Both proteins were co-incubated at 70 °C to ensure efficient domain swapping. Subsequently, we immobilized hCE on a Strep-Tactin® resin via its Strep-tag. If heterodimerization had occurred, we would have expected co-purification of the untagged hCC. However, using SDS-PAGE, we could not detect co-migration of hCC; solely hCE homodimer had bound (Fig. S4). This observation can probably be understood by the low sequence identity of only 30% between cystatin E and C. Sequence variations on the α1 helix, which is a critical connecting element, may result in steric clashes, thereby preventing heterodimerization. Inspection of the hCE and hCC monomer structures revealed, for example, a ^hCE^Ala29Phe^hCC^ variation that may be in steric conflict with Ser^114^-Gln-Leu^116^ (β5) on hCE. Thus, the affinity for homodimer formation is supposedly much higher than for heterodimerization. It might be possible to outcompete this affinity difference by having a large excess of hCC relative to hCE.

### Cystatin E forms amyloid fibrils

**Figure 5.**
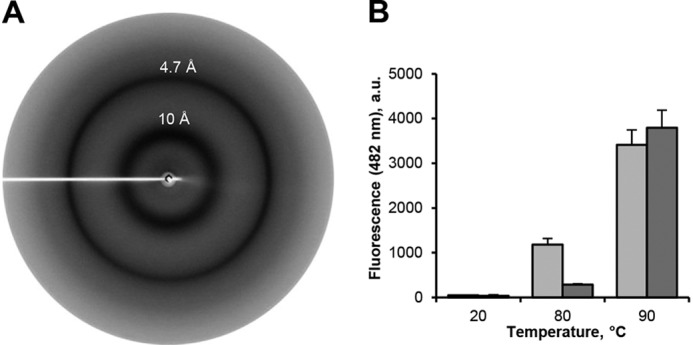
**Cystatin E forms cross-β structures.** *A*, X-ray diffraction experiments of insoluble hCE pellet revealed two rings at 10 and 4.7 Å resolution, which are characteristic for cross-β structures. *B*, monomeric (*light gray*) and dimeric (*dark gray*) hCE were incubated at 20, 80, and 90 °C for 10 min. Subsequently, binding of ThT was measured as an increase in fluorescence detected at 482 nm. *a.u.*, arbitrary units. *Error bars*, S.D.

Cystatin E domain swapping results in the formation of an energetically more favorable conformation. In the simplest situation, two domain-swapped monomers assemble to form a dimer. However, domain swapping is not restricted to dimerization, but in principle also allows the formation of multimers via concerted swapping reactions (chain reaction). The existence of such multimers was previously reported for stefin B and cystatin C and resulted in the assembly of highly ordered fibrils (26, 27, 30). Thermal denaturation curves of monomeric cystatin E revealed a first transition/partial unfolding between 60 and 70 °C, which corresponded to dimer formation (Fig. 1*C*). Additionally, we observed a second transition between 80 and 90 °C for both monomeric and dimeric hCE, pointing toward the presence of another conformational state like fibrils, analogous to cystatin C. To test this hypothesis, we incubated cystatin E at a temperature above the second transition point (90 °C). Thereby, we observed the formation of an insoluble protein pellet. To test whether this pellet contains misfolded, aggregated protein or rather structured amyloid fibrils, we performed X-ray diffraction experiments. Interestingly, we observed two diffraction maxima at 10 and 4.7 Å resolution, which are indicative of cross-β structures, which typically build up amyloid fibrils (Fig. 5*A*). Furthermore, we could confirm the presence of fibrils using thioflavin T, a dye specifically binding to amyloid structures (Fig. 5*B*). Even more, negative staining TEM experiments also revealed the presence of fibril structures (Fig. S5). In agreement with thermal denaturation curves, fibril formation is more efficient at 90 °C as compared with 80 °C because the energy barrier is only partially overcome at 80 °C (Figs. 1*C* and 5*B*).

### hCE fibrils contain functional protein

**Figure 6.**
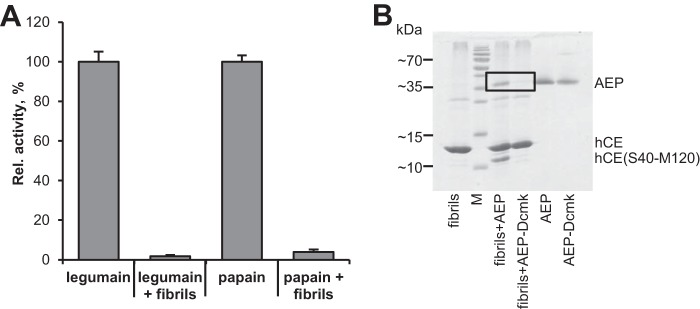
**hCE fibrils contain functional protein.** *A*, activity of legumain and papain was measured upon the addition of hCE amyloid fibrils in a fluorescent substrate assay using FR-AMC (papain) and Z-AAN-AMC (legumain) substrates. Control reactions contained only the respective enzyme. *B*, SDS-PAGE showing a co-precipitation assay of hCE fibrils and legumain. Insoluble hCE fibrils were incubated with active site–free legumain (*AEP*) and active site–blocked legumain (*AEP-Dcmk*). Subsequently, the insoluble fraction was harvested by centrifugation and loaded on SDS-PAGE. Control reactions contained fibrils only, AEP only, and active site–blocked legumain only. Whereas active site–free legumain bound to hCE-fibrils, active site–blocked legumain did not bind. Additionally, a band corresponding to P1-Asn^39^–processed hCE was observed (*hCE(S40-M120*); C-terminal cleavage product). *Error bars*, S.D.

Based on the assumption that fibril formation is a result of concerted domain-swapping reactions, it seemed possible that hCE fibrils contained structured, functional protein. Because domain swapping did not compromise the functionality of the legumain-binding site, we tested whether hCE fibrils can, like dimeric hCE, inhibit legumain activity. Indeed, upon co-incubation of legumain with hCE fibrils, we observed complete inhibition of the Ala-Ala-Asn-AMC substrate turnover (Fig. 6*A*). This observation was the first strong indicator of the presence of folded protein within hCE fibrils. However, inhibition might have also been caused by nonspecific precipitation mediated by insoluble hCE fibrils. To test whether the inhibition we observed is via specific interaction of hCE with the legumain active site, we performed a pulldown assay. Specifically, we co-incubated the insoluble hCE fibrils with active site–free and active site–blocked legumain variants. To block the active site, we used a covalent acetyl-Tyr-Val-Ala-Asp-chloromethylketone (acetyl-YVAD-cmk) inhibitor. hCE fibrils are insoluble and for that reason served as stationary phase to extract potentially bound legumain. If the interaction of legumain with hCE fibrils was specific, via its active site, we would have expected to see binding of active site–free legumain but not of the acetyl-YVAD-cmk–blocked variant. Indeed, we observed co-migration of WT legumain with hCE fibrils, whereas active site–blocked legumain remained in the soluble fraction (Fig. 6*B*). Furthermore, we observed specific processing of hCE fibrils at the Asn^39^ cleavage site on the legumain reactive center loop. Both results supported the presence of functional, folded protein within hCE fibrils. To test the structural integrity of the legumain exosite loop, we collected thermal denaturation curves of legumain pre-incubated with hCE fibrils. Analogous to monomeric and dimeric hCE, we observed a stabilizing effect of hCE fibrils on legumain at pH 6.5 (Fig. S6). The effect is not as pronounced as for the legumain–hCE (dimer) complex. However, this can be understood by the complexity of the fibrils.

### hCE fibrils inhibit papain(-like) enzymes

In principle, both monomeric and dimeric hCE are suitable building blocks for legumain-inhibitory hCE fibrils. However, the situation is different for papain. Only monomeric hCE is a functional papain inhibitor. Surprisingly, co-incubation of papain with hCE fibrils led to a complete inhibition of its enzymatic activity (Fig. 6*A*). Thus, hCE fibrils must contain, to some extent, monomeric hCE protein, which has not undergone domain swapping. Presumably, hCE fibrils are built up by domain-swapped monomers but are at the same time heterogeneously decorated with monomeric WT protein (Fig. 10).

### Dimeric hCE further converts to fibrils

**Figure 7.**
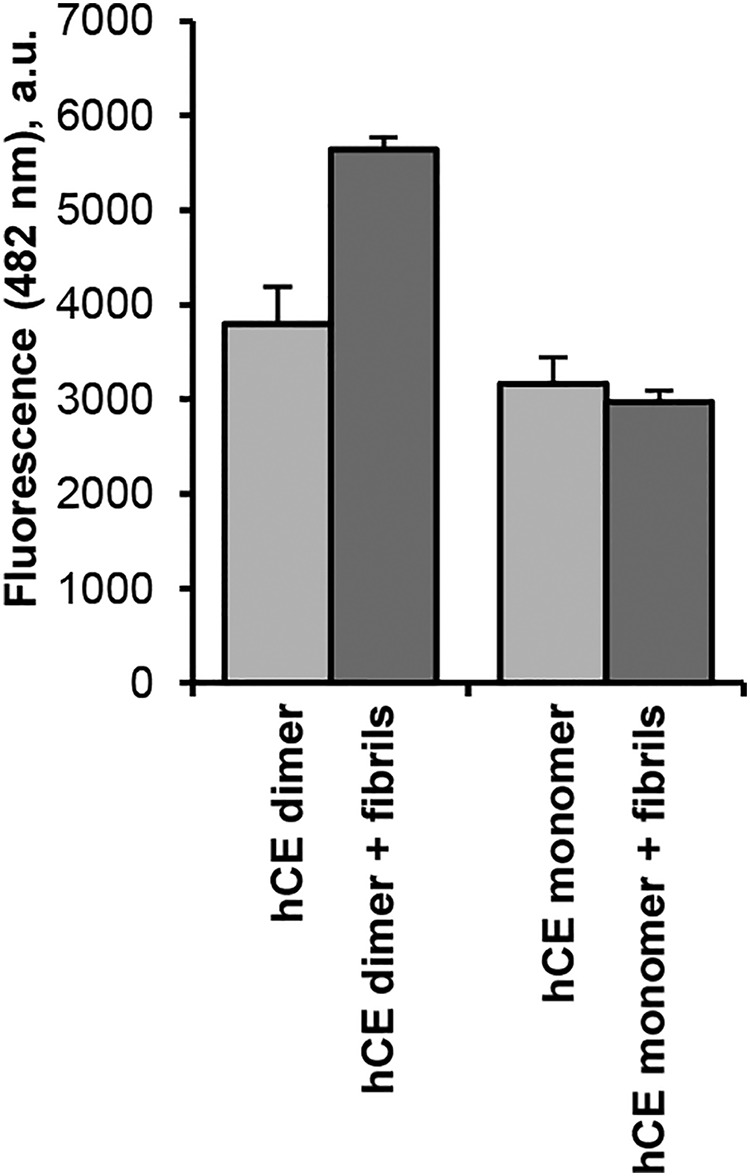
**hCE fibrils serve as a template for dimeric hCE to bind.** hCE monomer and dimer were incubated at 90 °C for 10 min in the presence (*dark gray*) and absence (*light gray*) of hCE fibrils. Subsequently, ThT binding was measured as an increase in fluorescence at 482 nm. Control reactions contained ThT only and fibrils only. *a.u.*, arbitrary units; *Error bars*, S.D.

Thermal denaturation experiments uncovered that dimeric hCE has a higher thermal stability as compared with monomeric hCE. However, both species showed a transition at *T* >80 °C, indicating that dimeric hCE can similarly be converted to amyloid fibrils when the energy burden is significantly lowered (*e.g.* by further increase in temperature) (Fig. 1*C*). Indeed, we found fibril formation of dimeric hCE upon incubation at 90 °C, as evidenced in a thioflavin T (ThT) test (Fig. 5*B*). Therefore, the dimer is not a dead end, but most likely a folding intermediate on the route to multimers. Because both monomeric and dimeric hCE are capable of fibril formation, both might potentially serve as a building block for fibrils. However, based on ThermoFluor experiments, we would rather suggest that the dimer is the critical intermediate. To study this hypothesis, we set up a fibril nucleation assay where we tested the ability of monomeric and dimeric hCE to extend preformed fibrils. Specifically, we co-incubated monomeric and dimeric hCE with purified fibrils at 90 °C for 10 min. Interestingly, we observed an increase in ThT signal for dimeric hCE spiked with fibrils as compared with the control reaction without preaddition of fibrils (Fig. 7). Thus, multimerization of dimeric hCE can be triggered by providing preformed fibrils as a “folding” template.

### Fibril formation is pH-dependent and incompatible with glycosylation

**Figure 8.**
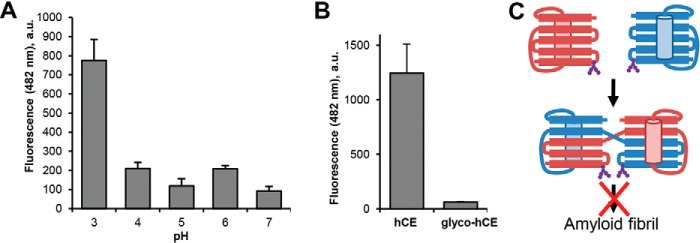
**hCE fibril formation is pH-dependent and incompatible with glycosylation.** *A*, monomeric hCE was incubated at the indicated pH values for 10 min. Subsequently, binding of ThT was measured as an increase in fluorescence at 482 nm. *B*, glyco-hCE produced in LEXSY (Jena Bioscience) and unglycosylated hCE produced in *E. coli* were incubated at 90 °C for 10 min. Subsequently, binding of ThT was measured by monitoring an increase in fluorescence at 482 nm. *C*, model of glycosylated hCE forming a dimer but not higher oligomers. *a.u.*, arbitrary units; *Error bars*, S.D.

Domain swapping is triggered by conformational destabilization of monomeric hCE. Besides heating, we could identify low pH as a trigger factor for hCE dimerization. Similarly, we tested the effect of acidic pH on fibril formation and thereby found an about 4-fold increase in ThT signal following incubation at pH 3.0 (Fig. 8*A*). Furthermore, ThermoFluor experiments revealed a reduction of the fibril transition temperature by ∼8 °C at pH 3.5 (Fig. 1*C*). Consequently, changing the pH environment will have an effect on the hCE oligomerization state. Along that line, we were also curious about the effect of glycosylation on hCE multimerization. Although dimerization is possible for the glycosylated variant, multimerization might be negatively affected because of potential steric conflicts. Indeed, we did not observe fibril formation of glycosylated cystatin E (glyco-hCE) upon incubation at elevated temperatures (Fig. 8, *B* and *C*). This suggested to us a regulatory function of glycosylation for hCE function.

## Discussion

**Figure 9.**
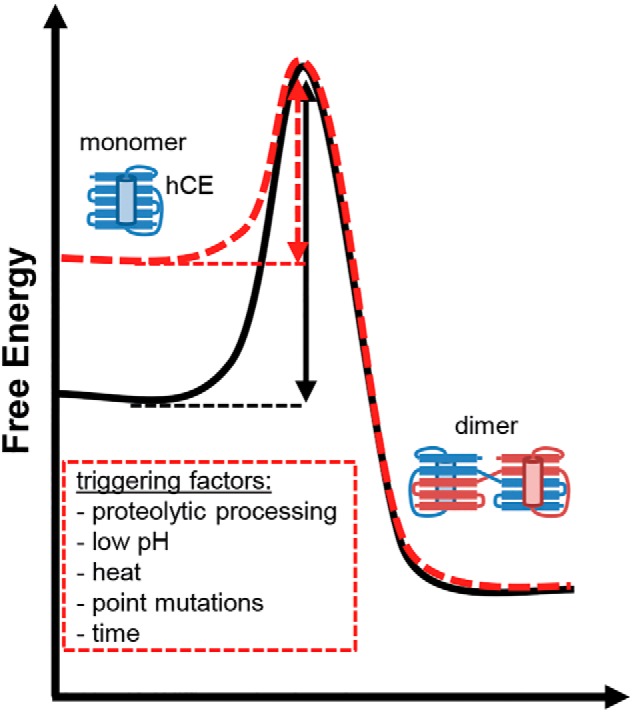
**Conversion of monomeric hCE to the dimer needs a source of energy.** The cystatin E monomer is a stable folding state that can convert to a dimeric state if a certain energy barrier is overcome (*black arrow*). The dimer has a higher thermal and fold stability as compared with the monomer. Factors reducing the energy barrier (*red arrow*) are time, pH, proteolytic processing, mutations, and temperature.

Cystatin E is an intrinsically stable protein with a melting temperature >60 °C. However, destabilization does not lead to hCE denaturation as would be the case for most other proteins, but results in the transition to an energetically more favorable conformation (Fig. 9). This second folding state, the hCE dimer, represents a structurally distinct conformation with increased conformational stability. The energy barrier that needs to be overcome to allow for domain swapping can be reduced by triggering factors. Within this study, we could identify N-terminal truncation, acidification, and heating as accelerators of dimerization (Fig. 9). Similarly, proteolytic processing has previously been reported for hCC, presumably catalyzed by leukocyte elastase, and led to faster dimerization and amyloid deposit formation (25). Along that line, a different but similar strategy of controlling the oligomerization state via proteolytic processing evolved in another member of the family 2 cystatins, cystatin F. Cystatin F harbors two additional cysteine residues, which form two intermolecular disulfide bonds and thereby connect two cystatin F monomers. That way, the papain-inhibitory site is blocked in an inhibitory latent dimeric cystatin F. However, the legumain-inhibitory site is freely accessible. Activation of cystatin F is mediated by disulfide reduction or proteolysis in the N-terminal region (19, 43). Although nothing is known so far about proteolytic processing of hCE *in vivo*, a similar event seems likely because of the flexible loop structure of the N-terminal region, which makes it prone to proteolytic processing. *In vitro*, we could previously demonstrate processing of hCC and hCE on their legumain RCL by legumain (17). Similarly, destabilization on this site may have a negative effect on the stability of monomeric and dimeric hCE. Interestingly, this cleavage is most efficient at acidic pH, where dimerization is also more easily achievable. Acidification is a common phenomenon in the brain of patients suffering from dementia, emphasizing its relevance for domain swapping and consequent aggregation (44, 45). Likewise, the L68Q hCC variant found in the cerebral fluid of patients suffering from Iceland disease has a higher tendency to form dimers and multimers. Replacement of the hydrophobic Leu^68^ by the hydrophilic glutamine causes local destabilization of the monomer, thereby also reducing the energy barrier for domain swapping (25). Such a destabilizing mutation has not yet been identified for hCE; however, comparable natural variations may exist.

Domain swapping results in a complete loss of hCE's papain-inhibitory activity and a 50% loss of the legumain binding capacity. Consequently, domain swapping of cystatins in general will lead to an increase in protease activity due to loss of the inhibitors. Domain swapping thus provides another strategy to regulate proteolytic activity and can cause dysregulation of protease activity under pathological conditions. Legumain can stabilize cystatin E in its monomeric and dimeric state by binding to the actual conformation and thereby preventing further domain swapping. However, papain(-like enzymes) can only bind and retain the cystatin E monomer.

**Figure 10.**
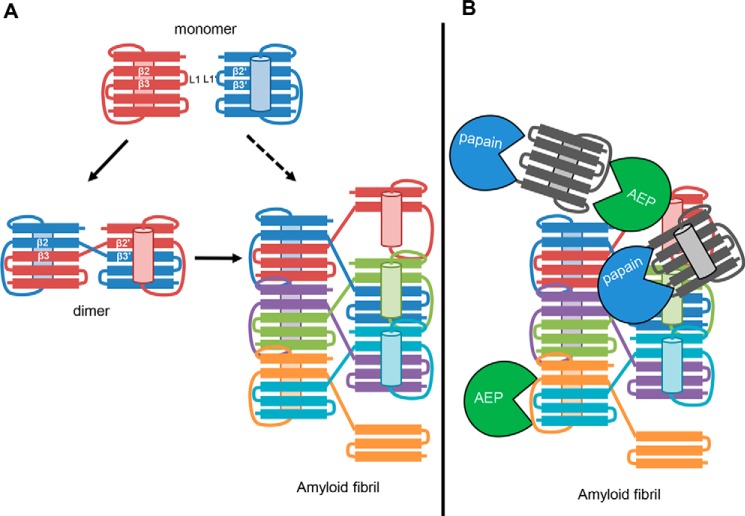
**Model of cystatin E oligomerization.** *A*, monomeric hCE can convert to a dimer upon mild conformational destabilization. Dimeric hCE can further convert to ordered oligomers potentially via concerted domain swapping reactions. The conversion from monomer to dimer and dimer to oligomer requires a certain energy barrier to be overcome. The energy barrier can be overcome by mild destabilization by low pH, N-terminal truncation, heat, and point mutations, among other factors. Presumably, dimeric hCE is a stable intermediate on the route to amyloid fibrils. Consequently, the conversion of monomeric hCE to fibrils very likely proceeds via the dimer. *B*, hCE fibrils are functional as legumain and papain inhibitors. For that reason, we suppose that they are heterogeneously composed of domain-swapped and monomeric subunits. The presence of hCE monomers (*dark gray units*) allows for the inhibition of papain-like enzymes. Inhibition of legumain is possible both with monomeric and domain-swapped hCE.

Although the conversion of monomeric to dimeric hCE is kinetically trapped, dimeric hCE can further convert to fibrils. Thus, domain swapping seems to be key for multimer formation. Whereas the addition of preformed fibrils to dimeric hCE led to an increase in ThT fluorescence, monomeric hCE was unaffected. This further confirms the relevance of the hCE dimer as a folding intermediate on the route to higher cystatin multimers. Additionally, it implies some prion-like behavior encoded in hCE. Because the hCE dimer has already been prone to domain swapping, it seems plausible that it is a better acceptor for a folding template than the monomer. However, it remains unclear whether monomeric hCE directly converts to fibrils if enough energy is supplied (1-step mechanism) or whether the dimer is formed first but immediately converted to fibrils (2-step mechanism; Fig. 10). A number of intermediate states have been described for other cystatins already, including a molten globule state or a tetramer (46, 47). Therefore, the full picture presumably is more complex and not restricted to one intermediate.

Amyloid proteins are generally considered to lose their native conformation while forming well-ordered cross-β structures (48). This allows them to assemble into large structures containing many copies of the same molecule. A prominent example of such a protein is the Aβ-peptide, which plays a central role in the pathology of Alzheimer's disease (49). Surprisingly, we could show that hCE amyloid fibrils contain biologically functional protein. Whereas the ability of hCE fibrils to inhibit legumain is in agreement with both domain-swapped and monomeric hCE, papain inhibition is only possible with monomeric hCE. Hence, we concluded that hCE amyloid fibrils contain domain-swapped hCE as a building block to fibril formation; however, they are heterogeneously decorated with monomeric hCE (Fig. 10). Domain swapping may not be 100% efficient, explaining how some monomeric protein remains to be integrated into fibrils. Survival of monomeric protein is presumably time-dependent. Consequently, longer incubation may result in less monomeric protein and less papain inhibition. Based on these observations, we assume that hCC fibrils also contain, to some extent, functional protease inhibitor.

Cystatin fibrils might serve as a binding platform to stabilize the pH-sensitive legumain and cathepsins in the extracellular environment, thereby increasing their lifetime. Because cystatins are reversible inhibitors, fibrils might provide a strategy of storing enzymes for later action. Although we did not observe heterodimerization between family 2 members, the formation of mixed fibrils seems possible. Protofibrils of hCE and hCC may assemble into mixed larger structures.

Unlike the family 1 cystatins, family 2 cystatins are frequently glycosylated. Human cystatin E harbors an *N*-glycosylation site on the L2 loop, which is compatible with dimerization but abolished fibril formation. From a structural perspective, modification on the L2 loop is in principle compatible with domain swapping but in steric conflict with the formation of large fibrils, where proteins need to be packed together tightly. Interestingly, both glycosylated and unglycosylated hCE were observed *in vivo* (*e.g.* in breast cancer cell lines or cystatin E–overexpressing HEK293 cells), suggesting different (pathophysiological) functions for both variants (10, 20, 37). Whereas glycosylated hCE will only be present as monomer or dimer, unglycosylated hCE can potentially also be converted to amyloid fibrils. Moreover, human cystatin C also harbors a predicted glycosylation site, but different from the one present in hCE. Cystatin C harbors an *O*-glycosylation site on its N-terminal region (Ser^2^). Glycosylation at this site will probably have an effect on dimerization, because this part of the cystatin molecule is directly involved in domain swapping. Indeed, there is indirect evidence from the literature that N-terminal glycosylation is incompatible with or at least restricting hCC dimerization (50). Whereas cystatin C may lose the *O*-glycosylation site by N-terminal proteolytic cleavage, the hCE *N*-glycosylation is not affected because of its location on the L2 loop (28, 51). However, (de)glycosidases might play a role as regulatory enzymes, switching glycosylated hCE to unglycosylated hCE. N-terminally truncated cystatin C was isolated from cystatin C amyloid deposits and results in accelerated formation of amyloid depositions (29).

Recently, cystatin E was also identified in the cerebrum (52). Thus, it is attractive to speculate about a potential role of domain-swapped cystatin E in neuronal function.

## Experimental procedures

### Preparation of proteins

WT hCE and hCC constructs lacking the N-terminal signal sequence were cloned into the pET22b(+) vector (Novagen) as described earlier (17). A truncated version of hCE (ΔhCE) was prepared using a forward primer carrying an NcoI restriction site (5′-ATGCCCATGGAACTGTCGCCCGACGACCCGCAGGTGC-3′) and a reverse primer carrying an XhoI restriction site (5′-ACGTCTCGAGCATCTGCACACAGTTGTGC-3′). The construct has residues Arg^4^–Leu^13^ deleted and, due to the employed cloning strategy, starts with the double mutation R14M/D15E. Full-length WT hCE, N-terminally truncated ΔhCE, and hCC had the native signal peptide replaced by the pelB leader sequence present on the pet22b(+) vector to allow expression to the *Escherichia coli* periplasmic space. During secretion, the signal peptide was removed, thereby liberating the new Arg^4^ N terminus of full-length WT hCE (hCC numbering), Met^14^ of the truncated ΔhCE variant, and Ser^1^ of hCC. Additionally, the expression constructs carried a C-terminal His_6_ tag for purification. Furthermore, another WT cystatin E construct carrying a C-terminal His_6_ tag followed by a Strep-tag was prepared. For that purpose, a WT cystatin E construct was used as a template, and the Strep-tag was introduced following a protocol based on the inverse-PCR method. As primers, we used TCAGTTCGAAAAGTGAGATCCGGCTGCTAACAAAGCCCGAAAGG (forward) and GGGTGTGACCAGTGGTGGTGGTGGTGGTGCTCGAGCATCTGC (reverse). Correctness of expression constructs was confirmed via DNA sequencing by Eurofins MWG Operon (Martinsried, Germany).

Cystatin E and C constructs were expressed in *E. coli* Bl21(DE3) cells. Briefly, the expression plasmid was transformed into Bl21(DE3) cells. For large-scale expression, cells were grown in 2-liter flasks filled with 600 ml of lysogeny broth medium (Carl Roth, Karlsruhe, Germany) supplemented with 100 μg/ml ampicillin at 37 °C with agitation at 220 rpm until an *A*_600_ of 0.8–1.0 was reached. Expression was induced at 25 °C by the addition of 1 mm isopropyl β-d-1-thiogalactopyranoside. After overnight expression, cells were harvested by centrifugation (10 min, 4000 rpm, 4 °C) and frozen. The periplasmic fraction containing recombinant proteins was extracted by cold osmotic shock. Briefly, frozen cell pellets of 1.8 liters of expression culture were gently resuspended in 150 ml of lysis buffer composed of 30 mm Tris, pH 7.5, and 20% sucrose and stirred for 20 min at ambient temperature. The lysate was centrifuged at 17,500 × *g* for 20 min at 4 °C. The supernatant containing soluble periplasmic proteins was batch-incubated with nickel-nitrilotriacetic acid Superflow resin (Qiagen, Hilden, Germany) for 30 min at 4 °C. Following a washing step using a buffer composed of 20 mm Tris, pH 7.5, 300 mm NaCl, and 10 mm imidazole, bound protein was eluted with washing buffer containing 250 mm imidazole. Elutions were concentrated using Amicon Ultra centrifugal filter units (molecular mass cutoff 3000 Da; Millipore) and further purified by SEC. The chromatography was run on an Äkta FPLC system equipped with a Superdex 75 10/300 GL column (GE Healthcare) which was equilibrated in a buffer composed of 20 mm citric acid, pH 5.5, and 100 mm NaCl. Glyco-hCE was produced using LEXSY following a protocol described previously (17). Human WT legumain was cloned, expressed, purified, and activated as described earlier (53).

### Determination of oligomerization state using SEC

To analyze the oligomerization state of different hCE variants after different treatments, 250 μl of sample were loaded on an S75 10/300 GL column equilibrated in a buffer composed of 20 mm citric acid, pH 5.5, and 100 mm NaCl. To test the effect of heating, WT cystatin E was incubated at 37, 60, 70, and 85 °C for 10 min at a concentration of 0.5 mg/ml in a buffer composed of 20 mm citric acid, pH 5.5, and 100 mm NaCl. After a 10-min incubation, the samples were chilled on ice for a further 20 min. N-terminally truncated cystatin E was incubated at 4 °C and 37 °C before injection, and WT cystatin C was incubated at 60 and 70 °C. Additionally, WT cystatin E was incubated at 60 °C in citric acid buffer at pH 3.0 to test the effect of pH on dimerization. To investigate the effect of glycosylation on dimerization, hCE produced in LEXSY was incubated at 80 °C for 10 min and subsequently analyzed by SEC.

To test the stoichiometry of the legumain–dimeric hCE complex, we incubated legumain (0.5 mg/ml) with dimeric hCE at a molar ratio of 1:1 (1 AEP and 1 hCE dimer) at pH 5.5 for 10 min on ice before injection of the sample on a S200 10/300 GL column. The hCE dimer was prepared by incubation of monomeric hCE at 80 °C for 10 min. Similarly, legumain was incubated with monomeric hCE at a molar ratio of 1:2 (1 AEP and 2 hCE monomers) for 10 min at pH 5.5. To calculate molar concentrations of monomeric and dimeric hCE, a molecular mass of 15 and 30 kDa, respectively, was assumed. Both samples contained equal amounts of cystatin E either in monomeric or dimeric state. For all samples investigated, fractions were collected and analyzed on SDS-PAGE.

### ThermoFluor assays

Thermal denaturation curves of different protein variants after different treatments were determined using the ThermoFluor method (54). Briefly, 1 mg/ml protein sample containing 50× SYPRO Orange (Invitrogen) was added in a 1:10 ratio to 22.5 μl of assay buffer composed of 50 mm citric acid pH 5.5/3.0 and 100 mm NaCl. Thermal denaturation curves were collected in a 7500 real-time PCR system (Applied Biosystems) from 20 to 95 °C. The fluorescence data were analyzed as described previously (55). Protein samples investigated were monomeric hCE and dimeric hCE, which was prepared by incubation of monomeric hCE at 80 °C for 10 min. To test the stability of AEP in complex with monomeric hCE and dimeric hCE, a complex was prepared at pH 5.5 by mixing AEP and inhibitor in a 1:1 molar ratio, assuming 30 kDa as the molecular mass of dimeric hCE. Stability of AEP only and in complex with hCE was assayed in a buffer composed of 50 mm MES, pH 6.5, and 100 mm NaCl. To test the effect of hCE fibrils on AEP stability, 1 μl of washed fibrils were added to the assay buffer (50 mm MES, pH 6.5, 100 mm NaCl) before the addition of AEP premixed with SYPRO Orange. Fibril preparation is described under “Co-precipitation assay.”

### Inhibition assays

Inhibition of WT legumain was tested in legumain assay buffer (50 mm citric acid, pH 5.5, 100 mm NaCl) containing 100 μm benzyloxycarbonyl-Ala-Ala-Asn-7-amino-4-methylcoumarin substrate (Z-AAN-AMC; Bachem). Assays were carried out in an Infinite M200 plate reader (Tecan). Briefly, the assay buffer was preincubated with 8 nm cystatin, followed by the addition of 4 nm legumain. Increase in fluorescence was measured at 460 nm upon excitation at 380 nm at 37 °C. Inhibition of papain (EC 3.4.22.2; Merck, Darmstadt, Germany) and recombinant human cathepsin S was assayed in the same assay buffer containing 100 μm Z-FR-AMC (Bachem). The assay buffer was preincubated with 100 nm cystatin, followed by the addition of 50 nm papain or cathepsin S, and fluorescence was similarly recorded at 460 nm. Dimeric cystatin E was prepared by incubation of monomeric cystatin E at 80 °C for 10 min. The sample was filtered to remove higher oligomers. Similarly, glycosylated cystatin E was also incubated at 80 °C for 10 min to generate the dimeric form. All experiments were carried out in triplicate.

### Determination of K_I_ values

Inhibition constants of monomeric and dimeric hCE toward legumain were determined in assay buffer composed of 50 mm citric acid, pH 5.5, 100 mm NaCl, and 0.05% Tween 20 using the Morrison equation for tight binding inhibitors (56). First, the *K_m_* value of legumain toward the Z-AAN-AMC substrate was determined in assay buffer containing 3–250 μm substrate. The reactions were started by the addition of 2 nm enzyme. The *K_m_* was calculated to be 52 μm under these assay conditions. In the next step, the assay buffer containing 100 μm Z-AAN-AMC substrate was preincubated with increasing concentrations of hCE ranging from 0.01 to 5 nm, and the reaction was started by the addition of 2 nm legumain. Fluorescence was monitored at 460 nm and 37 °C for 10 min. The velocity of substrate turnover was calculated as fluorescence units/s, and the data points were fit to the Morrison equation using GraphPad Prism version 7.0 (GraphPad Software, La Jolla, CA). To calculate molar concentrations of dimeric hCE, a molecular mass of 30 kDa was assumed. All measurements were performed in triplicates.

### Crystallization and structure solution of dimeric hCE

Dimeric hCE was prepared by incubation of 1 mg/ml WT hCE at 80 °C for 10 min. Subsequently, the protein sample was filtered to remove higher oligomers and subjected to SEC using a S75 10/300 GL column equilibrated in a buffer composed of 20 mm citric acid, pH 5.5, and 50 mm NaCl. Fractions containing dimeric protein were concentrated to ∼30 mg/ml final concentration using Amicon Ultra centrifugal filter units (molecular weight cutoff 10,000; Millipore). Initial crystallization screening was performed in a sitting-drop vapor diffusion setup. 0.4 μl of concentrated dimeric hCE were mixed with 0.4 μl of screen solution (Hampton Index HT or JBScreen Classic) and equilibrated with 60 μl of reservoir solution in 96-well Intelli Plates (Art Robbins Instruments) at 20 °C. After 1–2 weeks, crystals were observed in a condition composed of 20% PEG 8000 and 0.1 m Ches, pH 9.5. Crystals were harvested after stepwise addition of a cryo-protectant solution containing 22% PEG 8000, 0.1 m Ches, pH 9.5, and 20% glycerol and flash-frozen in liquid nitrogen. A native data set was collected at 100 K on beamline ID23-2 (ESRF, Grenoble) equipped with a Pilatus3_2 M detector to a resolution of 2.8 Å. 720 images were collected at a wavelength of 0.8726 Å at 0.5° oscillation range and 0.1-s exposure time.

Data processing was performed utilizing iMOSFLM (57) and SCALA from the CCP4 program suite (58). An initial search model was prepared using the crystal structure of monomeric hCE (PDB entry 4N6L) by removing the N-terminal region up to loop L1. This model was used as an initial search model for molecular replacement using PHASER (59). Repeated cycles of manual rebuilding in COOT (60) and refinement using phenix.refine (61) were carried out. The atomic coordinates and experimental structure factors have been deposited with the Protein Data Bank with accession code 6FK0. PyMOL (62) was used to create figures illustrating structures.

### Molecular modeling

A model of dimeric hCE in complex with legumain was created using Topmatch (63). Specifically, the crystal structure of the legumain–hCE complex (PDB entry 4N6O) served as a template to align the structure of dimeric hCE.

### Proteolysis and ligation assay

To test proteolysis at the P1-Asn^39^ residue on cystatin E, monomeric and dimeric cystatin E were incubated with legumain in a 1:2 molar ratio (1 legumain and 2 hCE; assuming a molecular mass of 15 kDa for both monomeric and dimeric hCE) at pH 4.0 at 37 °C until 80% turnover was observed, as judged by SDS-PAGE. Subsequently, the pH was shifted to 7.5, and the samples were incubated for 1 h at 37 °C. As control samples, we used legumain only and monomeric/dimeric hCE only. Progress of proteolysis and ligation was investigated after different time points via SDS-PAGE. Dimeric hCE was prepared from N-terminally truncated hCE.

### X-ray diffraction to test amyloid fibril formation

Monomeric hCE was incubated at 90 °C for 10 min at 20 mg/ml protein concentration. Subsequently, the insoluble fraction potentially containing amyloid fibrils was harvested by centrifugation (16,000 × *g*, 10 min, 4 °C). To wash off residual monomeric protein, the pellet was resuspended in double-distilled H_2_O and again harvested by centrifugation. The supernatant was discarded and washed another two times. The pellet was then mounted at the edge of a quartz glass capillary, and diffraction was assayed in house using a Bruker Microstar rotating anode generator mounted with a Mar345dtb detector.

### Testing pH dependence of fibril formation

To test for formation of amyloid fibrils, cystatin E was incubated at 80 °C for 10 min at a protein concentration of 10 mg/ml in a buffer composed of 50 mm citric acid and 100 mm NaCl (pH range 3–6) or 50 mm Tris and 100 mm NaCl (pH 7.0). Afterward, samples were put on ice for at least 10 min before testing fibril formation using a ThT test. Briefly, a ThT stock solution was prepared by dissolving 8 mg of ThT (Sigma-Aldrich) in 10 ml of PBS buffer. A working solution was prepared freshly each day by diluting the ThT stock solution 1:50 in PBS buffer. 24 μl of working solution were mixed with 1 μl of sample in a 386-well black polystyrene plate (Corning), and fluorescence was measured for 2 min at 25 °C with excitation at 440 nm and emission at 482 nm. Mean and S.D. values from three measurements were calculated.

### Testing the effect of glycosylation on fibril formation

To test the effect of L2 loop glycosylation of cystatin E on the formation of higher oligomers, we incubated cystatin E produced in LEXSY at 90 °C for 10 min at a concentration of 20 mg/ml in a buffer composed of 50 mm citric acid, pH 5.5, and 100 mm NaCl to induce oligomerization. Subsequently, the sample was incubated on ice for at least 10 min before analysis in a ThT assay.

### Testing inhibition of legumain and papain by hCE fibrils

Cystatin E fibrils were prepared by incubation of a 10 mg/ml sample at pH 3.0 and 80 °C for 10 min. The insoluble fraction was harvested by centrifugation (16,000 × *g*, 10 min); resuspended in washing buffer composed of 50 mm citric acid, pH 5.5, and 100 mm NaCl; and centrifuged again. This washing step was repeated three times. Afterward, the fibrils were resuspended in 30 μl of wash buffer. To test inhibition of legumain and papain, the substrate solution described before was preincubated with 1 μl of fibrils, and the reaction was started by the addition of the enzyme. In each case, the final reaction volume was 50 μl. Control reactions contained only the enzyme in substrate solution. All reactions were measured in triplicate.

### Co-precipitation assay

Cystatin E fibrils were prepared by incubation of monomeric hCE at a 20 mg/ml concentration at 90 °C for 10 min. The insoluble fraction was harvested by centrifugation (16,000 × *g*, 10 min). To remove any residual monomeric or dimeric protein contaminants, the pellet was washed three times with a buffer composed of 50 mm citric acid, pH 5.5, 100 mm NaCl. The final pellet was resuspended in 20 μl of wash buffer. 5 μl of fibrils were mixed with 5 μl of legumain (0.1 mg/ml final concentration) and incubated for 10 min at 20 °C. Afterward, the insoluble fraction was harvested by centrifugation and washed two times with washing buffer. Control reactions contained fibrils only, fibrils + AEP precomplexed with the covalent acetyl-YVAD-cmk, and legumain only.

### Testing fibril formation by the addition of preformed fibrils

Cystatin E fibrils were prepared and washed as described above. Dimeric hCE was prepared by incubation of monomeric protein at 80 °C for 10 min, followed by filtering. Monomeric and dimeric cystatin E (10 μl, 10 mg/ml) were supplemented with 1 μl of cystatin E fibrils and incubated at 90 °C for 10 min. Control samples contained fibrils only or cystatin E monomer/dimer only. Afterward, samples were incubated on ice for at least 10 min before setting up a ThT assay. The reaction was set up in a buffer composed of 50 mm citric acid, pH 5.5, and 100 mm NaCl.

### Cystatin E/C heterodimerization assay

To test heterodimerization of cystatins E and C, cystatin E carrying a C-terminal Strep-tag was co-incubated with cystatin C at 70 °C for 10 min. Following a further 10-min incubation on ice, the sample was loaded on a Strep-Tactin Sepharose resin (IBA GmbH, Göttingen, Germany) pre-equilibrated in wash buffer (100 mm Tris, pH 8.0, 150 mm NaCl). The flow-through was collected, and the resin was washed five times with wash buffer. Finally, bound protein was eluted by applying five times 100 μl of elution buffer containing 100 mm Tris, pH 8.0, 150 mm NaCl, and 2.5 mm desthiobiotin. Control samples were cystatin E incubated at 37 °C (to confirm binding of monomeric protein; positive control), cystatin E incubated at 70 °C (to confirm binding of dimeric protein), and cystatin C incubated at 70 °C (to test nonspecific binding of hCC; negative control). Fractions from different stages of purification were analyzed on SDS-PAGE.

### Transmission EM

Cystatin E amyloid fibrils were prepared from monomeric protein at a concentration of 20 mg/ml (in a buffer composed of 50 mm citric acid, pH 5.5, and 100 mm NaCl) by incubation at 90 °C for 10 min. The insoluble fraction was harvested and washed as described above. Subsequently, the pellet containing hCE amyloid fibrils was resuspended in buffer composed of 20 mm MES, pH 5.5, and 20 mm MgCl_2_. A 1:10 dilution was prepared and vortexed for 1 min. Subsequently, the solution was centrifuged for 10 min at room temperature, and the supernatant was removed carefully and fixed by the addition of 0.1% glutaraldehyde (final concentration) for 10 min. 10 μl of sample were incubated on Formvar carbon film-coated 400 mesh copper grids (Electron Microscopy Sciences, München, Germany) for 2 min and stained with 2% uranyl formate solution containing 25 mm NaOH for 40 s. TEM imaging was performed using a Philips CM 100 transmission microscope operating at 100 kV. Images were acquired using an AMT 4 × 4 megapixel CCD camera. Imaging was performed at ×28,500 magnification.

## Author contributions

E. D., J. C. H., and H. B. designed the experiments. K. H. and H. C. performed the TEM experiments. S. O. D. designed the X-ray experiment to test for fibril formation and contributed with valuable discussions. E. D. and J. C. H. performed all other experiments. E. D. and H. B. wrote the paper, and all authors reviewed the manuscript.

## Supplementary Material

Supporting Information

## Footnotes

2 The abbreviations used are:

AEP: asparaginyl endopeptidase
hCE: human cystatin E
hCC: human cystatin C
RCL: reactive center loop
LEL: legumain exosite loop
SEC: size exclusion chromatography
cmk: chloromethylketone
Z: benzyloxycarbonyl
AMC: 7-amino-4-methylcoumarin
glyco-hCE: glycosylated cystatin E
Ches: 2-(cyclohexylamino)ethanesulfonic acid
RMSD: root mean square deviation
ThT: thioflavin T
LEXSY: *Leishmania tarentolae* expression system
TEM: transmission electron microscopy.
