# Supplementary material for: Structural and functional analysis of cystatin E reveals enzymologically relevant dimer and amyloid fibril states
Source: J Biol Chem. 2018 Jul 2;293(34):13151–65. doi: 10.1074/jbc.RA118.002154 (PMC6109925; doi:10.1074/jbc.RA118.002154)
Supplement: Supporting Information [file supp_293_34_13151__index.html]

Structural and functional analysis of cystatin E reveals enzymologically relevant dimer and amyloid fibril states — Cystatin E transforms to multimers with distinct functions — Structural and functional analysis of cystatin E reveals enzymologically relevant dimer and amyloid fibril states — Cystatin E transforms to multimers with distinct functions — Supporting Information 

# Structural and functional analysis of cystatin E reveals enzymologically relevant dimer and amyloid fibril states

## Supporting Information

- Supporting Information - Supporting Figures after Revision
