# Supplementary material for: Structural and functional analysis of cystatin E reveals enzymologically relevant dimer and amyloid fibril states
Source: J Biol Chem. 2018 Jul 2;293(34):13151–65. doi: 10.1074/jbc.RA118.002154 (PMC6109925; doi:10.1074/jbc.RA118.002154)
Supplement: Supporting Information [file supp_RA118.002154_135634_2_supp_159746_pb1tzs.pdf]

SUPPORTING INFORMATION

Structural and functional analysis of cystatin E reveals enzymologically relevant dimer and amyloid fibril states

Authors: Elfriede Dall, Julia C. Hollerweger, Sven O. Dahms, Haissi Cui, Katharina Häußermann, and Hans Brandstetter.

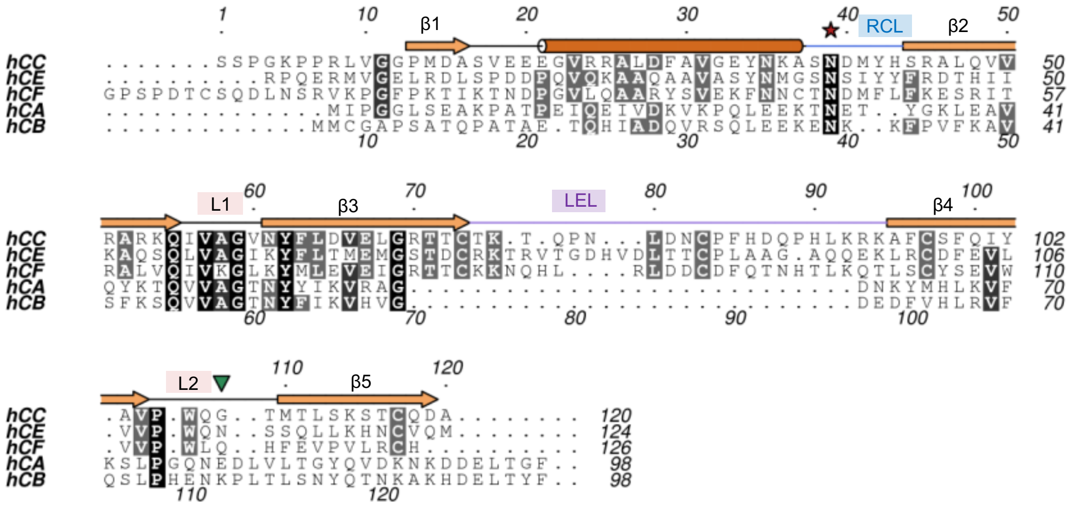

Figure S1. Structure-based sequence alignment of family 1 cystatins stefin A (hCA, P01040, pdb 3k9m) and stefin B (hCB, P04080, pdb 1stf), and family 2 cystatin C (hCC, P01034, pdb 3gax), E (hCE, Q15828, pdb 4n6l) and F (hCF, O76096, pdb 2ch9). The alignment was created using Topmatch [51] and visualized using Aline [52]. The top sequence numbering corresponds to hCC, the bottom numbering to hCE. Red star: P1-Asn39, green triangle: glyco-Asn106 in cystatin E.

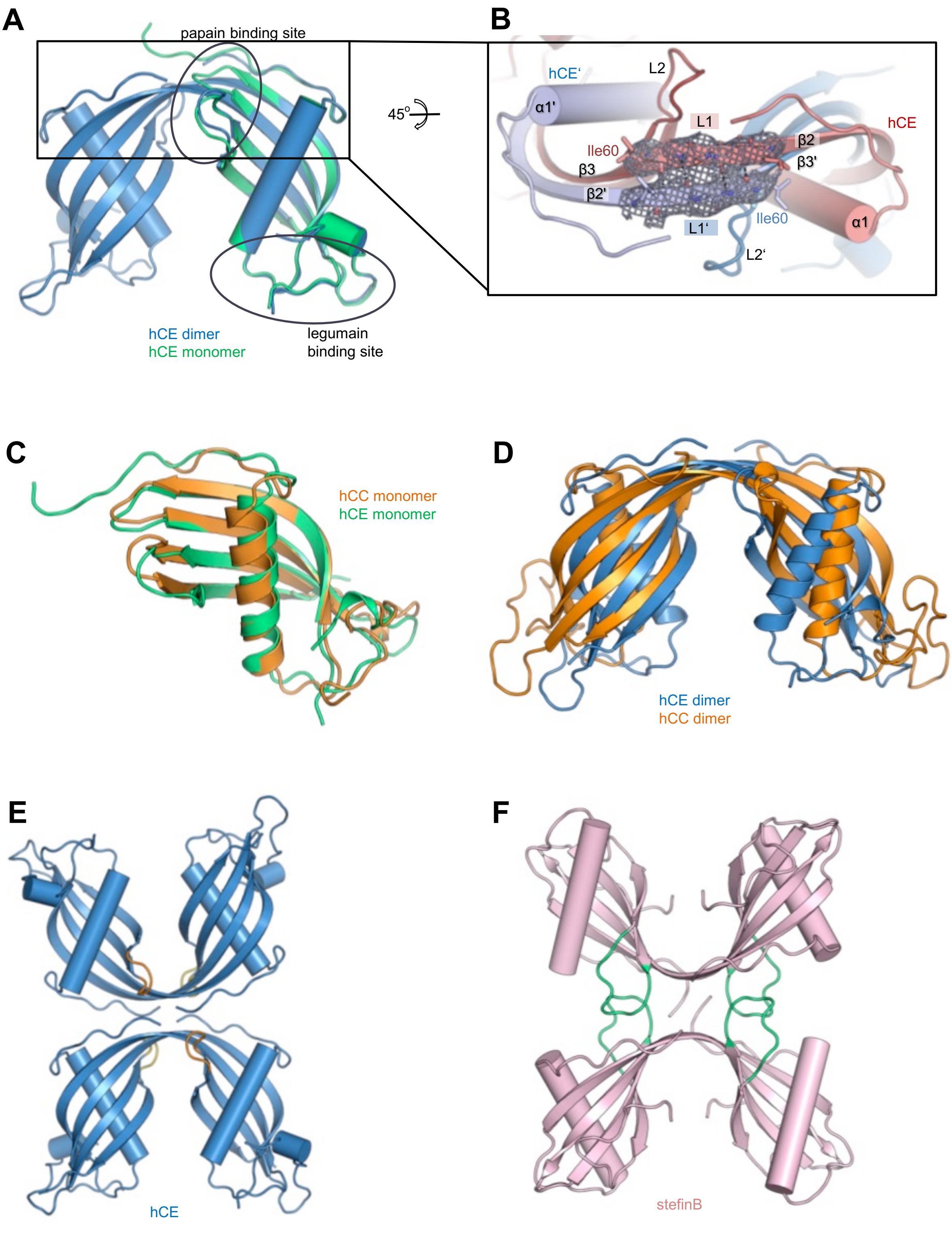

Figure S2. Structural comparison of cystatin dimers.

A) Superposition of the hCE dimer (blue) and monomer (green, pdb 4n6l). Papain and legumain binding sites are indicated by black circles.

B) Zoom-in view on the flexible hinge region formed by the former L1 and L1' loops. The N-terminal regions β1-α1-β2 up to the L1 loop that undergo domain swapping are colored in

light blue (hCE') and light red (hCE). The electron density map ($F_o - F_c$ omit map) defining the L1 loop (Leu56 – Ile 60) is contoured at 1.0 σ.

C) Superposition of monomeric hCE (green) and hCC (orange, pdb 3gax).

D) Superposition of dimeric hCE (blue) and dimeric hCC (orange, pdb 1g96) created using Topmatch [51].

E) hCE tetramer shown in cartoon representation. L2 loops are shown in yellow and orange.

F) The stefin B tetramer (pdb 2oct) in cartoon representation. L2 loops are shown in green.

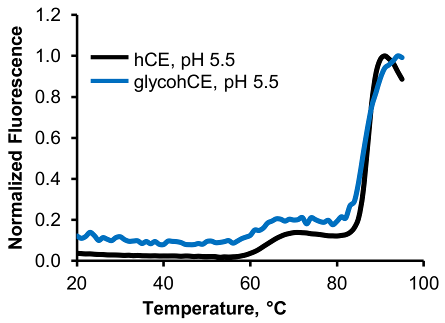

Figure S3. Glycosylated and unglycosylated hCE revealed similar melting temperatures. Thermofluor experiments of glycosylated hCE (glycohCE; blue line) produced in LEXSY and unglycosylated hCE produced in *E.coli* (black line) reveal a similar thermal unfolding behavior of both variants.

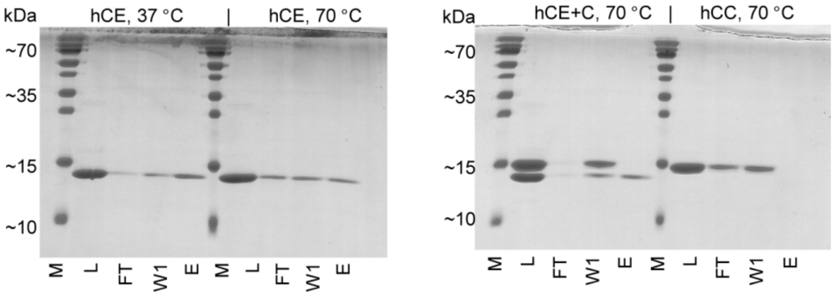

Figure S4. Hetero-dimerization between hCE and hCC was not observed under the investigated assay conditions.

To test whether hetero-dimerization is possible within the family 2 cystatins, cystatin E harboring a C-terminal strep-tag was mixed with cystatin C in a 1:1 molar ratio and incubated at 70 °C (a temperature where domain swapping is possible for both proteins). Following re-equilibration on ice, the sample was loaded onto Strep-Tactin® Sepharose beads. The beads were washed and bound protein was eluted by applying elution buffer supplemented with 2.5 mM desthiobiotin. Control experiments contained hCE incubated at 37 °C, hCE incubated at 70 °C and hCC incubated at 70 °C. Fractions of individual purifications steps were collected and subjected to SDS-PAGE. M: molecular weight marker, L: load, FT: flow through, W1: wash fraction 1, E: elution.

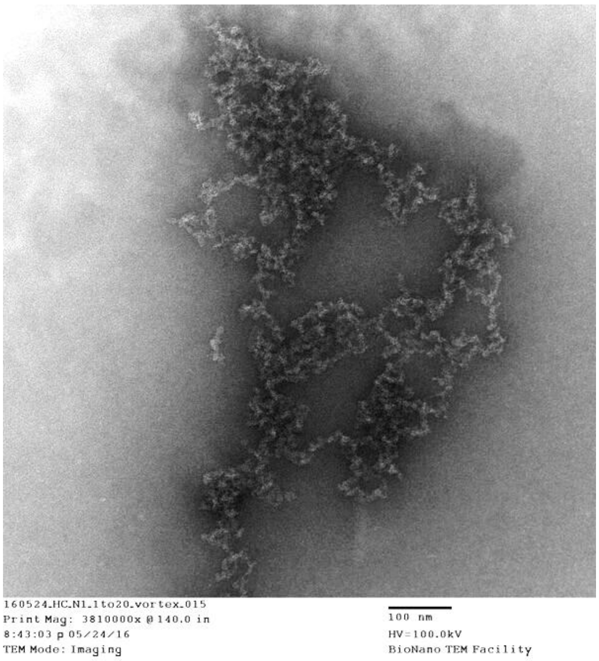

Figure S5. Negative staining TEM image of cystatin E amyloid fibrils. Fibrils were prepared from hCE monomer by incubation at 90 °C.

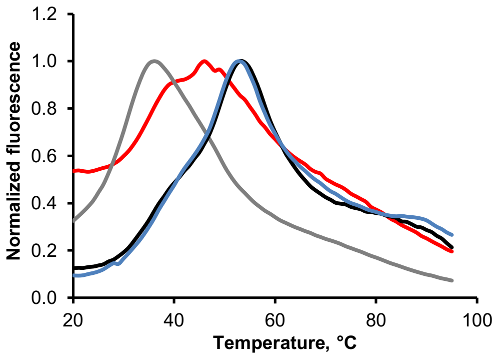

Figure S6. hCE amyloid fibrils have a stabilizing effect on legumain.

Thermal denaturation curves of legumain alone (grey curve), legumain pre-complexed with monomeric (black curve), dimeric hCE (blue curve) and fibrillary hCE (red curve) were collected at pH 6.5 using the thermofluor method. Complex formation led to an increase in thermal stability.

Table S1. Xray data collection and refinement statistics

	hCE dimer
**Data collection**	
Space group	$P222_1$
Cell dimensions	
a, b, c (Å)	31.6, 64.0, 147.7
α, β, γ (°)	90, 90, 90
Resolution (Å)[a]	58.7–2.9 (3.0–2.9)
R_{merge}	0.11 (1.56)
R_{pim}	0.04 (0.62)
CC (1/2) (%)	0.99 (0.80)
$I/\sigma I$	9.1 (1.3)
Completeness (%)	100.0 (100.0)
Redundancy	7.6 (7.2)
**Refinement**	
Resolution (Å)	58.7–2.9
No. reflections	7092
R_{work} / R_{free}	26.8/27.3
No. atoms	
Protein	1739
Ligand/ion	0
Water	0
Overall B-factor (Å^2)	131
R.m.s deviations	
Bond lengths (Å)	0.01
Bond angles (º)	1.33

The structure was determined from a single crystal.

[a] Highest resolution shell is shown in parentheses.
